# In Vitro Prebiotic and Anti-Colon Cancer Activities of Agar-Derived Sugars from Red Seaweeds

**DOI:** 10.3390/md19040213

**Published:** 2021-04-12

**Authors:** Eun Ju Yun, Sora Yu, Young-Ah Kim, Jing-Jing Liu, Nam Joo Kang, Yong-Su Jin, Kyoung Heon Kim

**Affiliations:** 1 Department of Biotechnology, Graduate School, Korea University, Seoul 02841, Korea; jdjddcld@korea.ac.kr (E.J.Y.); sora90715@korea.ac.kr (S.Y.); 2 Carl R. Woese Institute for Genomic Biology, University of Illinois at Urbana-Champaign, Urbana, IL 61801, USA; jingjing@sugarlogix.com; 3 School of Food Science and Biotechnology, Kyungpook National University, Daegu 41566, Korea; yakim@o.cnu.ac.kr (Y.-A.K.); njkang@knu.ac.kr (N.J.K.); 4 Department of Food Science and Human Nutrition, University of Illinois at Urbana-Champaign, Urbana, IL 61801, USA

**Keywords:** red seaweeds, agarose, agarotriose, 3,6-anhydro-l-galactose, prebiotics, anti-colon cancer activity

## Abstract

Numerous health benefits of diets containing red seaweeds or agar-derived sugar mixtures produced by enzymatic or acid hydrolysis of agar have been reported. However, among various agar-derived sugars, the key components that confer health-beneficial effects, such as prebiotic and anti-colon cancer activities, remain unclear. Here, we prepared various agar-derived sugars by multiple enzymatic reactions using an endo-type and an exo-type of β-agarase and a neoagarobiose hydrolase and tested their in vitro prebiotic and anti-colon cancer activities. Among various agar-derived sugars, agarotriose exhibited prebiotic activity that was verified based on the fermentability of agarotriose by probiotic bifidobacteria. Furthermore, we demonstrated the anti-colon cancer activity of 3,6-anhydro-l-galactose, which significantly inhibited the proliferation of human colon cancer cells and induced their apoptosis. Our results provide crucial information regarding the key compounds derived from red seaweeds that confer beneficial health effects, including prebiotic and anti-colon cancer activities, to the host.

## 1. Introduction

Modern lifestyles have caused social and economic concerns regarding health disorders such as chronic diseases and metabolic dysfunction on a global level [1,2]. The human gut microbiome is known to play crucial roles in various human diseases, including chronic disorders and metabolic disease [3]. Additionally, diet, considered to be one of the major factors causing such diseases, has been hypothesized to modulate the functionality of the human microbiome [4]. Thus, there is increasing interest in dietary fiber as prebiotics that can selectively stimulate the growth of probiotics, conferring health benefits.

Marine macroalgae are considered to be good sources of prebiotics owing to their abundance in carbohydrates [1,5]. Red macroalgae are known to have higher carbohydrate content and lower amounts of recalcitrant substrates to saccharification, such as insoluble fiber, compared to other types of marine macroalgae [6]. Agarose—the major component of red macroalgae cell walls—consists of alternating units of d-galactose and 3,6-anhydro-l-galactose (AHG), which are linked alternately with α-1,3- and β-1,4-glycosidic linkages [7]. Agarose is distinct among red macroalgal polysaccharides since it can reach the large intestine, where it is degraded, fermented, and metabolized by gut microorganisms after consumption [8].

Agarose and oligosaccharides from agarose, including agarooligosaccharides (AOSs) and neoagarooligosaccharides (NAOSs), have been reported to have prebiotic effects that can promote the growth of beneficial gut bacteria and increase levels of short-chain fatty acids (SCFAs) [9,10]. However, most previous studies used agarose extracts containing heterogeneous components with various degrees of polymerization (DPs). Therefore, it is difficult to specify the exact source and cause of such prebiotic effects.

In addition to prebiotic effects, red macroalgae are known to possess various biological functions, including anti-inflammatory and antioxidant activities [11,12]. Additionally, clinical trials suggested that daily intake of seaweeds, including brown and red seaweeds, is associated with a lower risk of colon, colorectal, and breast cancers in Asian people who frequently consume red or brown seaweeds [13,14,15]. Fucoidan has been reported to be a key component for the anticancer activity of brown macroalgae and can induce apoptosis of cancer cells [16,17,18]. However, very little is known regarding which components are responsible for the anticancer effects of red macroalgae.

In this study, we produced agarose-derived sugars with various DPs by multiple enzymatic reactions and investigated the prebiotic effect of each agar-derived sugar. We also tested the in vitro anti-colon cancer activity of agar-derived sugars, which could be released from red seaweeds diets. This study can provide basic information about the health benefits, such as prebiotic and anti-colon cancer effects, that can be obtained from dietary red seaweed.

## 2. Results and Discussion

### 2.1. Enzymatic Production of Agar-Derived Sugars with Various DPs

**Figure 1:**
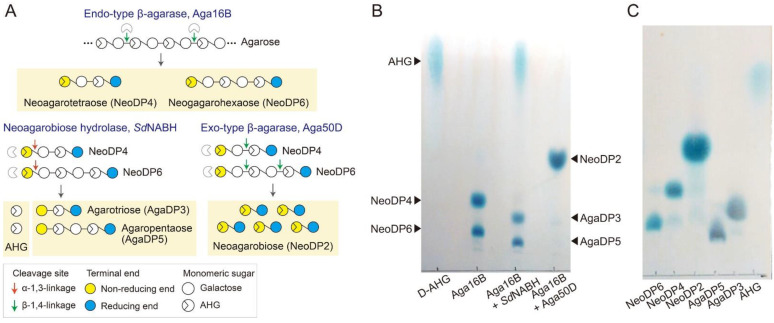
Enzymatic production of various agarose-derived sugars. (**A**) Schematic diagram illustrating the production of agar-derived sugars (i.e., AHG, NeoDP2, NeoDP4, NeoDP6, AgaDP3, and AgaDP5) by the enzymatic reactions of Aga16B, Aga50D, and *Sd*NABH. First, Aga16B produces NeoDP4 and NeoDP6 from agarose by the endolytic cleavage of agarose. *Sd*NABH subsequently produces AHG, AgaDP3, and AgaDP5 from the reaction products of Aga16B (mainly NeoDP4 and NeoDP6). Finally, Aga50D produces NeoDP2 from the reaction products of Aga16B. (**B**) Thin-layer chromatography analysis of the reaction products of Aga16B, Aga50D, and *Sd*NABH. (**C**) Purification of agar-derived sugars AHG, NeoDP2, NeoDP4, NeoDP6, AgaDP3, and AgaDP5 by gel-permeation chromatography of the reaction products of Aga16B, Aga50D, and *Sd*NABH.

Various agar-derived sugars were prepared by multiple enzymatic reactions using the purified recombinant enzymes Aga16B, Aga50D, and *Sd*NABH acting as an endo-type β-agarase, an exo-type β-agarase, and a neoagarobiose hydrolase, respectively (Figure 1A and Supplementary Materials Figure S1) [19,20,21]. Initially, the enzymatic liquefaction of agarose was performed using a thermostable endo-type β-agarase, Aga16B. Aga16B hydrolyzed agarose into NeoDP4 and NeoDP6 as the major reaction products, as described previously (Figure 1B) [21]. Then, the reaction products of Aga16B—which mainly comprised neoagarotetraose (NeoDP4) and neoagarohexaose (NeoDP6)—were hydrolyzed to AHG, agarotriose (AgaDP3), and agaropentaose (AgaDP5) by *Sd*NABH (Figure 1B) [19]. To produce neoagarobiose (NeoDP2), the reaction products of Aga16B—which mainly comprised NeoDP4 and NeoDP6—were further hydrolyzed to NeoDP2 by Aga50D (Figure 1B). After enzymatic production of agar-derived sugars from agarose, each sugar was purified by gel-permeation chromatography using a G-10 column (Figure 1C). Then, each purified sugar was used for testing in vitro prebiotic activity.

### 2.2. In Vitro Prebiotic Activity of Agar-Derived Sugars

**Figure 2:**
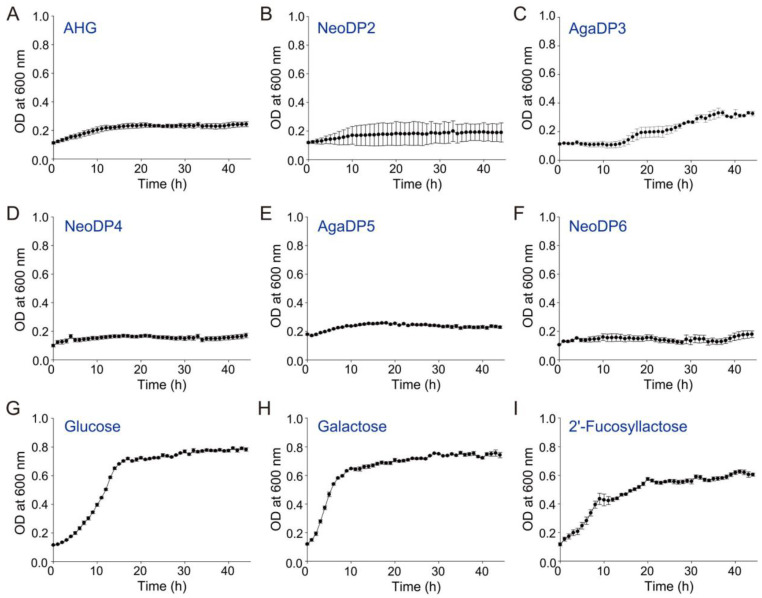
Screening of in vitro prebiotic effects of agar-derived sugars: (**A**) AHG, (**B**) NeoDP2, (**C**) AgaDP3, (**D**) NeoDP4, (**E**) AgaDP5, and (**F**) NeoDP6. *Bifidobacterium longum* ssp. *infantis* ATCC 15697 cells were cultured in synthetic de Man, Rogosa, and Sharpe broth supplemented with 5 g/L of each agar-derived sugar as a carbon source at 37 °C under anaerobic conditions. The positive control was 5 g/L (**G**) glucose, (**H**) galactose, or (**I**) 2′-fucosyllactose, which is a common prebiotic human milk oligosaccharide. During fermentation, the cell density was monitored by measuring optical absorbance at 600 nm using the Bioscreen C system.

A probiotic strain, *Bifidobacterium longum* ssp. *infantis* ATCC 15697, is known as a champion colonizer of the infant gut due to an extensive repertoire of bacterial genes that encode an array of glycosidases and oligosaccharide transporters not found in other gut bacteria [22]. The prebiotic effects of agar-derived sugars prepared in this study, AHG, NeoDP2, AgaDP3, NeoDP4, AgaDP5, and NeoDP6, were tested by examining their effects on the growth of *B. infantis* ATCC 15697 (Figure 2). Of the six agar-derived sugars tested in the present study, only AgaDP3 was utilized by *B. infantis* ATCC 15697 (Figure 2C). However, under AgaDP3, the cell growth of *B. infantis* ATCC 15697 was lower than that obtained under glucose, galactose, or 2′-fucosyllactose conditions (Figure 2G–I).

For the utilization of AgaDP3 by *B. infantis* ATCC 15697, it is presumed that *B. infantis* ATCC 15697 initially cleaves AgaDP3 into galactose and NeoDP2 by its β-galactosidase activity [23]. Galactose is utilized for the cell growth of *B. infantis* ATCC 15697. However, NeoDP2 is not utilized by *B. infantis* ATCC 15697 (Figure 2B). Therefore, incomplete utilization of AgaDP3 by *B. infantis* ATCC 15697 may cause the lower cell growth of *B. infantis* ATCC 15697 under the AgaDP3 condition (Figure 2C) compared to glucose, galactose, or 2′-fucosyllactose conditions (Figure 2G–I).

Meanwhile, the cell growth of *B. infantis* ATCC 15697 was not observed under the AgaDP5 condition (Figure 2E); this may be because although *B. infantis* ATCC 15697 can cleave AgaDP5 into galactose and NeoDP4 by its β-galactosidase activity, NeoDP4 is not utilized by *B. infantis* ATCC 15697 (Figure 2D). Therefore, galactose released from AgaDP5 may not be enough to support the cell growth of *B. infantis* ATCC 15697 as much as AgaDP3.

### 2.3. Fermentation of AgaDP3 by Bifidobacteria

**Figure 3:**
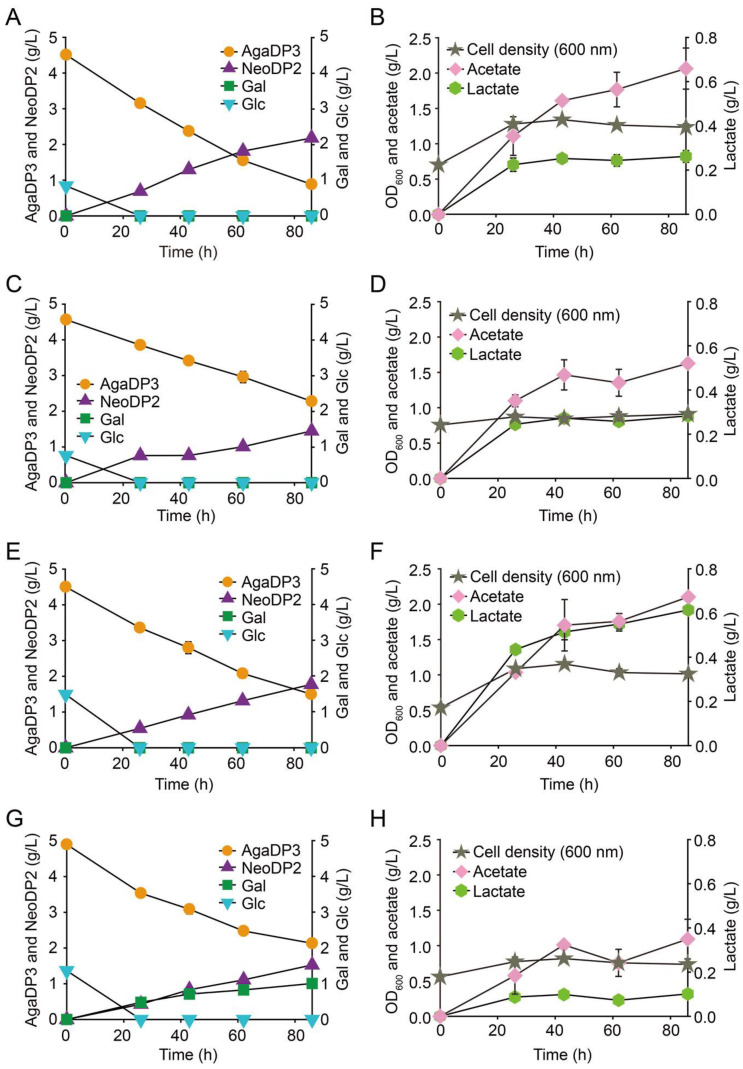
Fermentation of AgaDP3 by human milk oligosaccharide-utilizing *Bifidobacterium* strains. Fermentation profiles of AgaDP3 from various *Bifidobacterium* strains, namely, (**A**,**B**) *B. longum* ssp. *infantis* ATCC 17930, (**C**,**D**) *B. infantis* ATCC 15702, (**E**,**F**) *B. bifidum* DSM 20082, and (**G**,**H**) *B. kashiwanohense* DSM 21854. The *Bifidobacterium* strains were cultured in synthetic de Man, Rogosa and Sharpe broth supplemented with 5 g/L AgaDP3 as a carbon source at 37 °C under anaerobic conditions. During fermentation, cell density and the concentrations of AgaDP3, NeoDP2, galactose, glucose, acetate, and lactate were monitored.

Interestingly, it was reported that *B. infantis* ATCC 15697 possesses β-galactosidases (i.e., Bga42A and Bga2A), which are active on various human milk oligosaccharides (HMOs) belonging to type-1 and -2 isomers of HMOs [23]. These two enzymes are expected to be associated with the degradation of AgaDP3 by *B. infantis* ATCC 15697. This implies that not only *B. infantis* ATCC 15697, but also other HMO-utilizing *Bifidobacterium* strains might utilize AgaDP3. To verify this, the fermentation profiles of other probiotic HMO-utilizing *Bifidobacterium* strains—*B. infantis* ATCC 17930 [24], *B. infantis* ATCC 15702 [24], *Bifidobacterium kashiwanohense* DSM 21854 [25], and *Bifidobacterium bifidum* DSM 20082 [24]—were evaluated under the AgaDP3 condition. We found that among the four different probiotic HMO-utilizing *Bifidobacterium* strains, *B. infantis* ATCC 17930, *B. infantis* ATCC 15702, and *B. kashiwanohense* DSM 21854 metabolized AgaDP3 as a carbon source (Figure 3A‒H). These results indicated the possible high potential of AgaDP3 from red seaweeds as a prebiotic due to the utilization of AgaDP3, not only by *B. infantis* ATCC 15697, but also by other *Bifidobacterium* strains tested in this study.

The modes of action for the degradation of AgaDP3 exhibited by *B. infantis* ATCC 17930, *B. infantis* ATCC 15702, and *B. kashiwanohense* DSM 21854 can be explained as follows: AgaDP3 is initially transported into the cytosol of cells, and the intracellular β-galactosidases belonging to GH42 or GH2 cleave AgaDP3 into galactose and NeoDP2. Then, non-fermentable NeoDP2 is exported from the cells (Figure 3A–F).

In contrast, the degradation of AgaDP3 by *B. bifidum* DSM 20082 on AgaDP3 was different from that of the other strains (Figure 3G). *B. bifidum* DSM 20082 hydrolyzed AgaDP3 into galactose and NeoDP2 extracellularly, probably using secreted β-galactosidases [26]. However, *B. bifidum* DSM 20082 did not consume galactose (Figure 3G), which is fermentable by this strain [27]. Therefore, it was presumed that NeoDP2 accumulated in the culture medium might have inhibited the consumption of galactose (Figure 3G).

Next, the stability of AgaDP3 in simulated gastric fluid was tested because oral administration of AgaDP3 might lead to its decomposition in the stomach before reaching the intestine. As the incubation time increased, AgaDP3 was partially degraded to agarobiose and galactose due to the cleavage of the α1,3-glycosidic bond of AgaDP3 under low pH conditions (Supplementary Materials Figure S2) [28]. However, it was found that more than 80% of AgaDP3 was stably maintained in simulated gastric fluid at 37 °C for 3 h (Supplementary Materials Figure S2).

### 2.4. In Vitro Anti-Colon Cancer Activity of AHG

**Figure 4:**
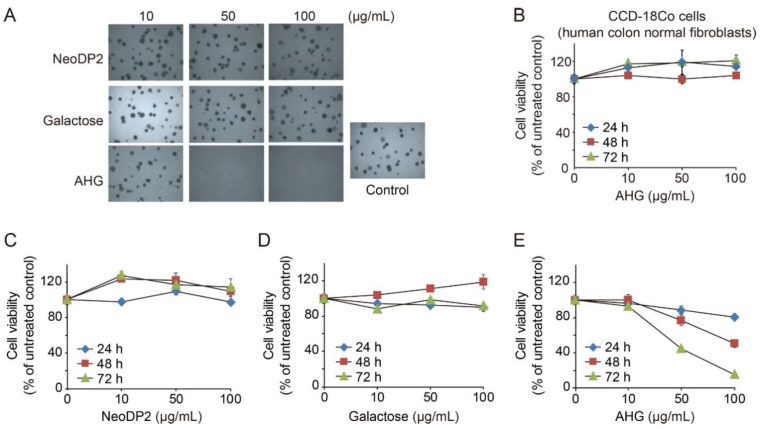
In vitro anti-colon cancer activity of AHG. (**A**) Inhibitory effects of NeoDP2, galactose, and AHG on the formation of HCT-116 human colon cancer cell colonies. (**B**) Effects of AHG on the viability of CCD-18Co cells, human colon normal fibroblasts. Error bars represent means ± SD. (**C**–**E**) Effects of NeoDP2 (**C**), galactose (**D**), and AHG (**E**) on the viability of HCT-116 cells. Error bars represent means ± SD.

We investigated the in vitro anti-colon cancer activity of monomeric and dimeric sugars, AHG, galactose, and NeoDP2, which can be released from agarose or AOSs by the actions of agarolytic marine or gut bacteria [29,30]. Our results showed that among the three different sugars, only AHG significantly inhibited the growth of HCT-116 human colon cancer cells (Figure 4A) and reduced their viability (Figure 4E). NeoDP2 and galactose neither inhibited cell growth (Figure 4A) nor reduced HCT-116 cell viability (Figure 4C,D). Intriguingly, AHG did not reduce the viability of CCD-18Co cells (human colon normal fibroblasts) (Figure 4B). Therefore, among the three different monomeric and dimeric sugars generated from agarose degradation (i.e., AHG, NeoDP2, and galactose), only AHG exhibited in vitro anti-colon cancer activity. Notably, AHG selectively inhibits the growth of HCT-116 human colon cancer cells but does not inhibit the growth of CCD-18Co cells, suggesting that AHG has a high potential for the development of anti-colon cancer agents.

**Figure 5:**
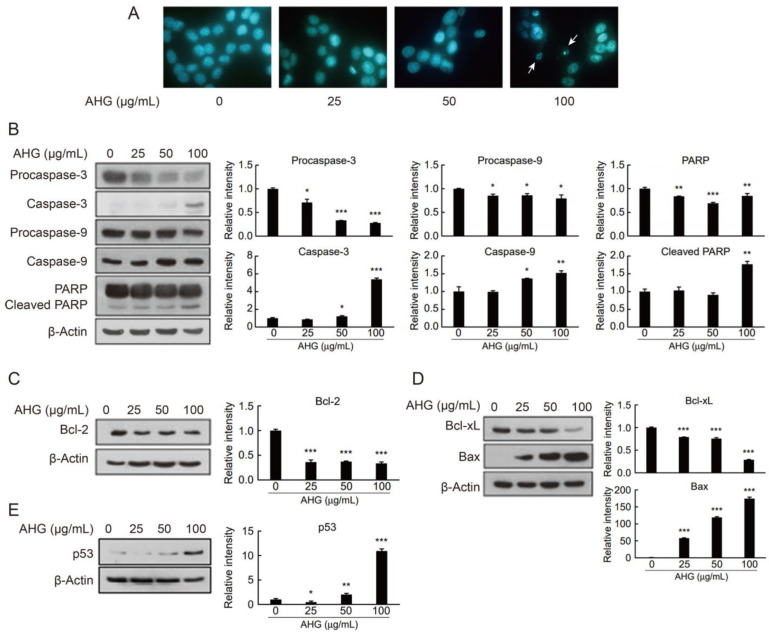
Induction of apoptosis in HCT-116 human colon cancer cells by AHG. (**A**) 4′,6-Diamidino-2-phenylindole (DAPI) staining of HCT-116 cells treated with AHG. (**B**–**E**) Western blot results showing the effects of AHG on expression of apoptosis-related proteins in cancer cells: procaspase-3 (**B**), caspase-3 (**B**), procaspase-9 (**B**), caspase-9 (**B**), poly(ADP-ribose) polymerase (PARP) (**B**), cleaved PARP (**B**), Bcl-2 (**C**), Bcl-xL (**D**), Bax (**D**), and p53 (**E**). β-actin was used as a control for all Western blot experiments. The relative intensities were quantified using an Image J program and normalized by the intensity of β-actin. Data are mean ± SD relative to the untreated group. *, *p* < 0.05; **, *p* < 0.01; ***, *p* < 0.001 vs. untreated control.

DAPI staining results showed that upon HCT-116 cell treatment with AHG, apoptotic bodies typically observed in apoptosis were formed in the colon cancer cells (Figure 5A). Apoptosis is a form of programmed cell death (PCD) that is controlled by numerous apoptotic proteins [31]. Apoptosis is an energy-dependent process that requires the activation of a group of cysteine proteases (caspases). Initiator caspases-8, -9, or -10 activate executioner caspase-3. In AHG-treated HCT-116 cells, the expression levels of the active forms of caspase-3 and caspase-9 were both elevated (Figure 5B). Once caspases are activated, the execution phase of apoptosis is triggered. As expected, one of the execution pathway proteins, poly (ADP-ribose) polymerase (PARP), was cleaved and activated (Figure 5B). Apoptosis is a complex process that is controlled and regulated by B-cell lymphoma (Bcl)-2 family proteins. Accordingly, AHG reduced the expression levels of anti-apoptotic proteins Bcl-2 and Bcl-xL and enhanced the expression of the pro-apoptotic protein Bax in HCT-116 cells (Figure 5C,D). Interestingly, the tumor suppressor protein P53, which is involved in apoptosis, was also induced by AHG (Figure 5E).

Taken together, we prepared agar-derived sugars with various DPs by multiple enzymatic reactions to investigate the physiological activities, especially prebiotic and anti-colon cancer activities, of each agar-derived sugar. We demonstrated in vitro prebiotic activity of AgaDP3 and in vitro anti-colon cancer activity of AHG. Our results suggest that marine macroalgae-derived oligosaccharides can be utilized as prebiotics. Moreover, the monosaccharides constituting marine macroalgae such as AHG can be utilized as potential anti-cancer agents.

## 3. Materials and Methods

### 3.1. Preparation of Agar-Derived Sugars by Enzymatic Hydrolysis of Agarose

Various agar-derived sugars were produced by multiple enzymatic reactions using Aga16B, Aga50D, and *Sd*NABH. The recombinant purified enzymes were prepared following the previously described methods [19,20,21]. To produce NAOSs, including NeoDP4 and NeoDP6, 100 mL of a reaction mixture comprising 1 mg of a thermostable endo-type β-agarase, Aga16B [21], and 1% (*w/v*) agarose in 20 mM Tris-HCl (pH 7.0) was incubated at 50 °C and 200 rpm for 12 h. To produce AHG, AgaDP3, and AgaDP5, 2.5 mg of neoagarobiose hydrolase, *Sd*NABH [19], was added to 100 mL of the reaction products of Aga16B containing NeoDP4 and NeoDP6. The reaction mixture was then incubated at 30 °C and 200 rpm for 12 h. To produce NeoDP2, 10 mg of an exo-type β-agarase, Aga50D [20], was added to 100 mL of the reaction products of Aga16B, and the reaction mixture was incubated at 30 °C and 200 rpm for 12 h. All enzymatic reactions were terminated by heating the reaction samples in boiling water for 3 min. After terminating enzymatic reactions, the reaction products obtained from each sample were identified by thin-layer chromatography (TLC).

### 3.2. Purification of Agar-Derived Sugars by Size-Exclusion Chromatography

To purify the agar-derived sugars—that is, AHG, NeoDP2, NeoDP4, NeoDP6, AgaDP3, and AgaDP5—by gel-permeation chromatography, the reaction products obtained from the enzymatic reactions using Aga16B, Aga50D, and *Sd*NABH were loaded onto a Sephadex G-10 column (Sigma-Aldrich, St. Louis, MO, USA) equilibrated with water. Each 1.5 mL fraction was obtained by elution with water as the mobile phase, and only the fractions containing each sugar were collected; this was confirmed by TLC.

### 3.3. TLC Analysis

TLC analysis of the enzymatic reaction products was conducted on a silica gel 60 plate (Merck, Burlington, MA, USA), and the plate was developed with an *n*-butanol–ethanol–water mixture (3:1:1, *v/v/v*) for 1 h. The plate was then dried and visualized using a solution comprising 10% (*v/v*) sulfuric acid and 0.2% (*w/v*) 1,3-dihydroxynaphthalene (Sigma-Aldrich) in ethanol at 90 °C for 1 min.

### 3.4. Screening of In Vitro Prebiotic Effects of Agar-Derived Sugars

To screen for in vitro prebiotic effects of the agar-derived sugars—that is, AHG, NeoDP2, NeoDP4, NeoDP6, AgaDP3, and AgaDP5—we cultured one of the most common probiotic bacteria, *B. infantis* ATCC 15697 in 200 µL of synthetic de Man, Rogosa, and Sharpe (sMRS) broth supplemented with 5 g/L of each agar-derived sugar as a carbon source. The sMRS broth was composed of 10 g/L peptone, 5 g/L yeast extract, 2 g/L anhydrous dipotassium phosphate, 5 g/L anhydrous sodium acetate, 2 g/L tribasic ammonium citrate, 0.2 g/L magnesium sulfate heptahydrate, 0.05 g/L manganese (II) sulfate, 1 mL/L polysorbate 80, 0.5 g/L cysteine, and 5 g/L of a carbon source [32]. The positive control was 5 g/L glucose, galactose, or 2′-fucosyllactose, which is a common prebiotic human milk oligosaccharide. During fermentation, cell growth was monitored by measuring optical density at 600 nm (OD_600_) using a Bioscreen C system (Labsystems, Helsinki, Finland).

### 3.5. Fermentation of AgaDP3 by Bifidobacteria

Five *Bifidobacterium* strains—namely, *B. infantis* ATCC 15697, *B. infantis* ATCC 17930, *B. infantis* ATCC 15702, *B. bifidum* DSM 20082, and *B. kashiwanohense* DSM 21854—were cultured in de Man, Rogosa and Sharpe (MRS; Sigma-Aldrich) or sMRS broth. The *Bifidobacterium* strains were cultured in a chamber with an anaerobic atmosphere comprising 90% N_2_ and 10% CO_2_, or 90% N_2_, 5% H_2_, and 5% CO_2_ (Airgas, Radnor, PA, USA) at 37 °C. During fermentation, cell growth was monitored by measuring OD_600_.

### 3.6. High-Performance Liquid Chromatography Analysis

A high-performance liquid chromatography (HPLC) system (1200 Series, Agilent Technologies, Santa Clara, CA, USA) equipped with an H^+^ (8%) column (Rezex ROA-Organic Acid; Phenomenex, Torrance, CA, USA) and a refractive index (RI) detector were used for HPLC analysis. The flow rate of the 0.005 N H_2_SO_4_ mobile phase was set at 0.6 mL/min, and the column and RI detector temperatures were set at 50 °C. For the quantitative analysis of AHG, NeoDP2, and AgaDP3, authentic standards of d-AHG, NeoDP2, and AgaDP3 were purchased from Carbosynth (Compton, Berkshire, UK) (Supplementary Materials Figure S3).

### 3.7. High-Performance Anion-Exchange Chromatography with Pulsed Amperometric Detection Analysis

A high-performance anion-exchange chromatography with pulsed amperometric detection (HPAEC-PAD) on a Dionex ICS-5000 system (Thermo Fisher Scientific, Waltham, MA, USA) equipped with a Dionex CarboPac PA100 column (250 mm × 2 mm; Thermo Fisher Scientific) was used for the quantitative analysis of NeoDP4, NeoDP6, and AgaDP5 [33]. At a flow rate of 0.25 mL/min, a gradient comprising the following mobile phases at 25 °C were used: (A) double-distilled water, (B) 0.1 M sodium hydroxide, (C) 0.1 M sodium hydroxide with 0.2 M sodium acetate, and (D) 0.25 M sodium hydroxide with 1 M sodium acetate. Before running samples, the column was washed with 100% D for 15 min, a linear gradient to 100% C for 10 min, and subsequently to 90% A and 10% B. Then, the column was equilibrated with 90% A and 10% B for 20 min. After sample injection, the following gradient was applied: 0 to 10 min, isocratic 90% A and 10% B; 10 to 20 min, linear to 100% B; 20 to 65 min, linear to 50% B and 50% C; 65 to 80 min, linear to 100% C; and 80 to 90 min, linear to 100% D. Cellotetraose (Sigma-Aldrich), cellopentaose (Sigma-Aldrich), and cellohexaose (Sigma-Aldrich) were used as standards for the quantitative analysis of NeoDP4, AgaDP5, and NeoDP6, respectively (Supplementary Materials Figure S3).

### 3.8. Stability Test of AgaDP3 on Simulated Gastric Fluid

To test the stability of AgaDP3 in gastric fluid, 1 g/L (*w/v*) AgaDP3 was incubated in a simulated gastric fluid comprising 0.2% (*w/v*) sodium chloride in 0.7% (*v/v*) hydrochloric acid (pH 1.11) at 37 °C for 4 h. Residual amounts of AgaDP3 were measured by HPLC every hour.

### 3.9. In Vitro Anti-Colon Cancer Activity Test of AHG Using Soft Agar Assay

To evaluate the inhibitory effects of NeoDP2, galactose, and AHG on colony formation by human colon cancer cells, HCT-116 cells (Korean Cell Line Bank, Seoul, Republic of Korea) were used. The HCT-116 cells were cultured on a 6-well soft agar plate for two weeks at 37 °C in the presence of various concentrations of NeoDP2, galactose, and AHG. Cell colonies were observed under a Nikon phase-contrast microscope (Nikon, Tokyo, Japan).

### 3.10. In Vitro Anti-Colon Cancer Activity Test of AHG by Using Cell Viability Assay

To estimate the effects of NeoDP2, galactose, and AHG on the viability of HCT-116 cells, cell proliferation was determined using a 3-(4,5-dimethylthiazol-2-yl)-2,5-diphenyltetrazolium bromide (MTT) assay [34]. One hundred microliters of Roswell Park Memorial Institute (RPMI) 1640 medium supplemented with 10% fetal bovine serum (FBS) was inoculated with 3 × 10^4^ cells/mL of HCT-116 cells in each well of a 96-well plate. NeoDP2, galactose, and AHG were added to the culture medium at a final concentration of 10, 50, or 100 µg/mL. After culturing for 72 h, 20 µL of the MTT solution was added to each well. The cells were then incubated for 2 h at 37 °C in a 5% CO_2_ incubator, and the optical absorbance of the cell culture was measured at 570 nm. CCD-18Co cells (American Type Culture Collection, Manassas, VA, USA) at 5 × 10^4^ cell/mL in Eagle’s Minimum Essential Medium (EMEM) with 10% FBS were cultured at 100 µL per well in a 96-well plate.

### 3.11. In Vitro Anti-Colon Cancer Activity Test of AHG by 4′,6-Diamidino-2-Phenylindole Staining

Apoptotic cell death was determined morphologically using the fluorescent nuclear dye 4′,6-diamidino-2-phenylindole (DAPI). HCT-116 cells (3 × 10^4^/mL) were cultured in a 6 cm dish for 24 h, treated with AHG (0–100 µg/mL) for 72 h, and then fixed with 100% ethanol for 30 min. The fixed cells were washed with PBS and stained with the DNA-specific fluorochrome DAPI (1 mg/mL). After 10 min of incubation, the cells were washed with PBS, deposited onto microscope slides, and observed under a fluorescent microscope (Nikon) to detect apoptotic characteristics.

### 3.12. In Vitro Anti-Colon Cancer Activity Test of AHG by Western Blot

To investigate the possible mechanism by which AHG inhibits the growth of HCT-116 cells, Western blot analysis of changes in expression of genes involved in apoptosis was carried out.

To measure the expression levels of certain apoptosis-related proteins—that is, procaspase-3, caspase-3, procaspase-9, caspase-9, PARP, cleaved PARP, Bcl-2, p53, Bcl-xL, and Bax—HCT-116 cells were seeded in a 60 mm dish and incubated at 37 °C and 5% CO_2_ for 24 h using RPMI 1640 medium supplemented with 10% FBS. After 24 h, the medium was replaced, but this time AHG at concentrations of 25, 50, and 100 µg/mL was included. The cells were then incubated under the same conditions described above, until at least approximately 80% of the bottom of each well was confluent with cells.

Western blot analysis was performed after incubation for 5 days. The cells were washed twice with phosphate-buffered saline, and the cells attached to the bottom of the well were collected using a lysis buffer comprising 20 mM Tris-HCl (pH 7.5), 150 mM NaCl, 1 mM ethylenediaminetetraacetic acid disodium salt (Na_2_EDTA), 1 mM ethylene glycol-bis(β-aminoethyl ether)-*N*,*N*′,*N*′,*N*-tetraacetic acid (EGTA), 1% Triton X-100, 2.5 mM sodium pyrophosphate, 1 mM β-glycerophosphate, 1 mM Na_3_VO_4_, 1 μg/mL leupeptin, 1 mM phenylmethylsulfonyl fluoride (PMSF), and a protease inhibitor cocktail. The resuspended solution containing the cells was then centrifuged at 18,407× *g* for 10 min at 4 °C. After obtaining each supernatant, the protein concentration was quantified using a protein assay kit (Bio-Rad Laboratories, Hercules, CA, USA).

Each protein prepared (20–40 µg) was degenerated and separated by SDS-PAGE, then transferred to a polyvinylidene difluoride (PVDF) membrane presoaked in methanol at 100 V for 2 h. The PVDF membrane was then immersed for 2 h in TBST solution (a mixture of Tris-buffered saline (TBS) and polysorbate 20) containing 5% skim milk to block non-specific protein binding sites. Next, the following antibodies purchased from Cell Signaling Technology (Danvers, MA, USA) were used to determine expression levels of the indicated proteins: rabbit polyclonal anti-human caspase-3, rabbit polyclonal anti-human caspase-9, rabbit polyclonal anti-human PARP, rabbit polyclonal anti-human Bcl-2, rabbit polyclonal anti-human Bcl-xL, rabbit polyclonal anti-human Bax, rabbit monoclonal anti-human p53, and mouse monoclonal anti-β-actin. The antibodies described above were diluted in TBST containing 5% skim milk at a ratio of 1:1000, and the reaction was carried out at 4 °C overnight. Anti-rabbit IgG (Santa Cruz Biotechnology, Dallas, TX, USA) and anti-mouse IgG (Santa Cruz Biotechnology) were used as secondary antibodies at a dilution of 1:5000, and the reactions were performed at 25 °C for 2 h. The PVDF membranes were then washed 4 times with TBST and reacted for 1–3 min with an enhanced chemiluminescence (ECL) substrate (Amersham, Little Chalfont, BM, UK). The membranes were pre-sensitized to X-ray film in a dark room to determine expression levels of apoptosis-related proteins present in each sample. The band intensities were quantified using an Image J from NIH (Bethesda, MD, USA) and normalized by the intensity of the β-actin, a loading control. The protein expression levels were expressed as relative intensities (fold change) for the untreated group. Single statistical comparisons were performed using a Student’s *t*-test. The data represented three independent experiments that gave similar results.

## 4. Conclusions

We prepared various agar-derived sugars through multiple enzymatic reactions and demonstrated the in vitro prebiotic and anti-colon cancer activities of agar-derived sugars. Specifically, AgaDP3 was consumed by HMO-utilizing probiotic bifidobacteria such as *B. infantis* and *B. kashiwanohense*. We also elucidated for the first time that AHG exhibits in vitro anti-colon cancer activity. Specifically, AHG significantly inhibited the proliferation of colon cancer cells, and induced apoptosis of such cells. Therefore, we conclude that AgaDP3 and AHG are key compounds that confer various health benefits, especially prebiotic and anti-colon cancer effects, to the host. Our results provide crucial information regarding potential key compounds derived from red seaweeds that confer health-beneficial effects, such as prebiotic and anti-colon cancer activities in humans; it can be applied to discover new functional food ingredients derived from dietary fibers.

## Data Availability

The datasets used and/or analyzed during the current study are available from the corresponding author upon reasonable request.

## Supplementary Materials

The following are available online at https://www.mdpi.com/1660-3397/19/4/213/s1, Figure S1. Sodium dodecyl sulfate–polyacrylamide gel electrophoresis analysis of the purified recombinant proteins of Aga16B, Aga50D, and *Sd*NABH. Lanes: M, protein markers; 1‒3, (1) Aga16B, (2) Aga50D, and (3) *Sd*NABH purified by His-tag affinity chromatography. Figure S2. Stability test of AgaDP3 in the presence of simulated gastric fluid. (A) AgaDP3 was incubated with simulated gastric fluid comprising 0.2% (*w/v*) sodium chloride in 0.7% (*v/v*) hydrochloric acid at 37 °C for 3 h. The concentration of AgaDP3 was monitored using HPLC. (B) Overlaid HPLC chromatograms profiling the partial degradation of AgaDP3 during incubation. Figure S3. Calibration curves of purified agar-derived sugars produced from agarose by the enzymatic reactions of Aga16B, Aga50D, and *Sd*NABH. (A–C) Calibration curves of AHG, NeoDP2, and AgaDP3 for quantitative analyses by HPLC. (D–F) Calibration curves for cellotetraose, cellopentaose, and cellohexaose for quantitative analyses of NeoDP4, AgaDP5, and NeoDP6, respectively, by HPAEC-PAD.

## References

[1] de Borba Gurpilhares D., Cinelli L.P., Simas N.K., Pessoa A., Sette L.D. (2019). Marine prebiotics: Polysaccharides and oligosaccharides obtained by using microbial enzymes. Food Chem..

[2] Maheshwari G., Sowrirajan S., Joseph B. (2019). β-Glucan, a dietary fiber in effective prevention of lifestyle diseases–An insight. Bioact. Carbohydr. Diet. Fibre.

[3] Lozupone C., Faust K., Raes J., Faith J.J., Frank D.N., Zaneveld J., Gordon J.I., Knight R. (2012). Identifying genomic and metabolic features that can underlie early successional and opportunistic lifestyles of human gut symbionts. Genome Res..

[4] Sonnenburg J.L., Bäckhed F. (2016). Diet–microbiota interactions as moderators of human metabolism. Nature.

[5] Wells M.L., Potin P., Craigie J.S., Raven J.A., Merchant S.S., Helliwell K.E., Smith A.G., Camire M.E., Brawley S.H. (2017). Algae as nutritional and functional food sources: Revisiting our understanding. J. Appl. Phycol..

[6] Yun E.J., Kim H.T., Cho K.M., Yu S., Kim S., Choi I.-G., Kim K.H. (2016). Pretreatment and saccharification of red macroalgae to produce fermentable sugars. Bioresour. Technol..

[7] Knutsen S., Myslabodski D., Larsen B., Usov A. (1994). A modified system of nomenclature for red algal galactans. Bot. Mar..

[8] Shang Q., Jiang H., Cai C., Hao J., Li G., Yu G. (2018). Gut microbiota fermentation of marine polysaccharides and its effects on intestinal ecology: An overview. Carbohydr. Polym..

[9] Ramnani P., Chitarrari R., Tuohy K., Grant J., Hotchkiss S., Philp K., Campbell R., Gill C., Rowland I. (2012). In vitro fermentation and prebiotic potential of novel low molecular weight polysaccharides derived from agar and alginate seaweeds. Anaerobe.

[10] Zhang N., Mao X., Li R., Hou E., Wang Y., Xue C., Tang Q.-J. (2017). Neoagarotetraose protects mice against intense exercise induced fatigue damage by modulating gut microbial composition and function. Mol. Nutr. Food Res..

[11] Chen H., Yan X., Zhu P., Lin J. (2006). Antioxidant activity and hepatoprotective potential of agaro-oligosaccharides in vitro and in vivo. Nutr. J..

[12] Enoki T., Okuda S., Kudo Y., Takashima F., Sagawa H., Kato I. (2010). Oligosaccharides from agar inhibit pro-inflammatory mediator release by inducing heme oxygenase 1. Biosci. Biotechnol. Biochem..

[13] Kim J., Lee J., Oh J.H., Chang H.J., Sohn D.K., Shin A., Kim J. (2020). Associations among dietary seaweed intake, c-MYC rs6983267 polymorphism, and risk of colorectal cancer in a Korean population: A case–control study. Eur. J. Nutr..

[14] Minami Y., Kanemura S., Oikawa T., Suzuki S., Hasegawa Y., Nishino Y., Fujiya T., Miura K. (2020). Associations of Japanese food intake with survival of stomach and colorectal cancer: A prospective patient cohort study. Cancer Sci..

[15] Yang Y.J., Nam S.J., Kong G., Kim M.K. (2010). A case-control study on seaweed consumption and the risk of breast cancer. Br. J. Nutr..

[16] Aisa Y., Miyakawa Y., Nakazato T., Shibata H., Saito K., Ikeda Y., Kizaki M. (2005). Fucoidan induces apoptosis of human HS-sultan cells accompanied by activation of caspase-3 and down-regulation of ERK Pathways. Am. J. Hematol..

[17] Ale M.T., Maruyama H., Tamauchi H., Mikkelsen J.D., Meyer A.S. (2011). Fucoidan from *Sargassum* sp. and *Fucus vesiculosus* reduces cell viability of lung carcinoma and melanoma cells in vitro and activates natural killer cells in mice in vivo. Int. J. Biol. Macromol..

[18] Kim E.J., Park S.Y., Lee J.Y., Park J.H. (2010). Fucoidan present in brown algae induces apoptosis of human colon cancer cells. BMC Gastroenterol..

[19] Ha S., Lee S., Lee J., Kim H., Ko H.-J., Kim K., Choi I.-G. (2011). Crystal structure of a key enzyme in the agarolytic pathway, α-neoagarobiose hydrolase from *Saccharophagus degradans* 2-40. Biochem. Biophys. Res. Commun..

[20] Kim H.T., Lee S., Lee D., Kim H.S., Bang W.G., Kim K.H., Choi I.-G. (2010). Overexpression and molecular characterization of Aga50D from *Saccharophagus degradans* 2-40: An exo-type β-agarase producing neoagarobiose. Appl. Microbiol. Biotechnol..

[21] Kim J.H., Yun E.J., Seo N., Yu S., Kim D.H., Cho K.M., An H.J., Kim J.H., Choi I.-G., Kim K.H. (2017). Enzymatic liquefaction of agarose above the sol-gel transition temperature using a thermostable endo-type β-agarase, Aga16B. Appl. Microbiol. Biotechnol..

[22] Underwood M.A., German J.B., Lebrilla C.B., Mills D.A. (2015). *Bifidobacterium longum* subspecies *infantis*: Champion colonizer of the infant gut. Pediatr. Res..

[23] Yoshida E., Sakurama H., Kiyohara M., Nakajima M., Kitaoka M., Ashida H., Hirose J., Katayama T., Yamamoto K., Kumagai H. (2011). *Bifidobacterium longum* subsp. *infantis* uses two different β-galactosidases for selectively degrading type-1 and type-2 human milk oligosaccharides. Glycobiology.

[24] Garrido D., Ruiz-Moyano S., Lemay D.G., Sela D.A., German J.B., Mills D.A. (2015). Comparative transcriptomics reveals key differences in the response to milk oligosaccharides of infant gut-associated bifidobacteria. Sci. Rep..

[25] James K., Bottacini F., Contreras J.I.S., Vigoureux M., Egan M., Motherway M.O., Holmes E., van Sinderen D. (2019). Metabolism of the predominant human milk oligosaccharide fucosyllactose by an infant gut commensal. Sci. Rep..

[26] Kitaoka M. (2012). Bifidobacterial enzymes involved in the metabolism of human milk oligosaccharides. Adv. Nutr..

[27] Yun E.J., Liu J.-J., Lee J.W., Kwak S., Yu S., Kim K.H., Jin Y.-S. (2019). Biosynthetic routes for producing various fucosyl-oligosaccharides. ACS Synth. Biol..

[28] Yang B., Yu G., Zhao X., Jiao G., Ren S., Chai W. (2009). Mechanism of mild acid hydrolysis of galactan polysaccharides with highly ordered disaccharide repeats leading to a complete series of exclusively odd-numbered oligosaccharides. FEBS J..

[29] Pluvinage B., Grondin J.M., Amundsen C., Klassen L., Moote P.E., Xiao Y., Thomas D., Pudlo N.A., Anele A., Martens E.C. (2018). Molecular basis of an agarose metabolic pathway acquired by a human intestinal symbiont. Nat. Commun..

[30] Yu S., Yun E.J., Kim D.H., Park S.Y., Kim K.H. (2020). Dual agarolytic pathways in a marine bacterium, *Vibrio* sp. strain EJY3: Molecular and enzymatic verification. Appl. Environ. Microbiol..

[31] Elmore S. (2007). Apoptosis: A review of programmed cell death. Toxicol. Pathol..

[32] Barrangou R., Altermann E., Hutkins R., Cano R., Klaenhammer T.R. (2003). Functional and comparative genomic analyses of an operon involved in fructooligosaccharide utilization by *Lactobacillus acidophilus*. Proc. Natl. Acad. Sci. USA.

[33] Wefers D., Dong J., Abdel-Hamid A.M., Paul H.M., Pereira G.V., Han Y., Dodd D., Baskaran R., Mayer B., Mackie R.I. (2017). Enzymatic mechanism for arabinan degradation and transport in the thermophilic bacterium *Caldanaerobius polysaccharolyticus*. Appl. Environ. Microbiol..

[34] Freimoser F.M., Jakob C.A., Aebi M., Tuor U. (1999). The MTT [3-(4,5-dimethylthiazol-2-yl)-2,5-diphenyltetrazolium bromide] assay is a fast and reliable method for colorimetric determination of fungal cell densities. Appl. Environ. Microbiol..

