# Supplementary material for: In Vitro Prebiotic and Anti-Colon Cancer Activities of Agar-Derived Sugars from Red Seaweeds
Source: Mar Drugs. 2021 Apr 12;19(4):213. doi: 10.3390/md19040213 (PMC8070132; doi:10.3390/md19040213)
Supplement: Supplementary file 1 [file marinedrugs-19-00213-s001.pdf]

Supplementary Materials

In vitro prebiotic and anti-colon cancer activity of agar-derived sugars from red seaweeds

Eun Ju Yun[1,2,†], Sora Yu[1,†], Young-Ah Kim[3], Jing-Jing Liu[2,4], Nam Joo Kang[3], Yong-Su Jin[2,4,*], and Kyoung Heon Kim[1,*]

[1]Department of Biotechnology, Graduate School, Korea University, Seoul 02841, Republic of Korea

[2]Carl R. Woese Institute for Genomic Biology, University of Illinois at Urbana-Champaign, Urbana, Illinois 61801, USA

[3]School of Food Science and Biotechnology, Kyungpook National University, Daegu 41566, Republic of Korea

[4]Department of Food Science and Human Nutrition, University of Illinois at Urbana-Champaign, Urbana, Illinois 61801, USA

†These authors contributed equally to this work.

*Correspondence: khekim@korea.ac.kr (K.H.K.); ysjin@illinois.edu (Y.S.J.)

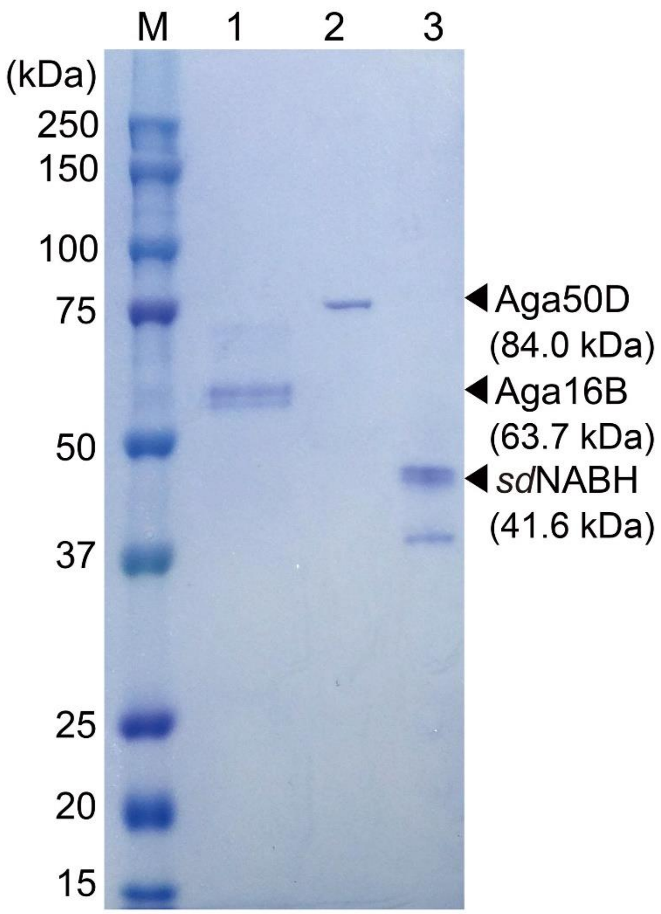

Figure S1. Sodium dodecyl sulfate–polyacrylamide gel electrophoresis analysis of the purified recombinant proteins of Aga16B, Aga50D, and *Sd*NABH. Lanes: M, protein markers; 1–3, (1) Aga16B, (2) Aga50D, and (3) *Sd*NABH purified by His-tag affinity chromatography.

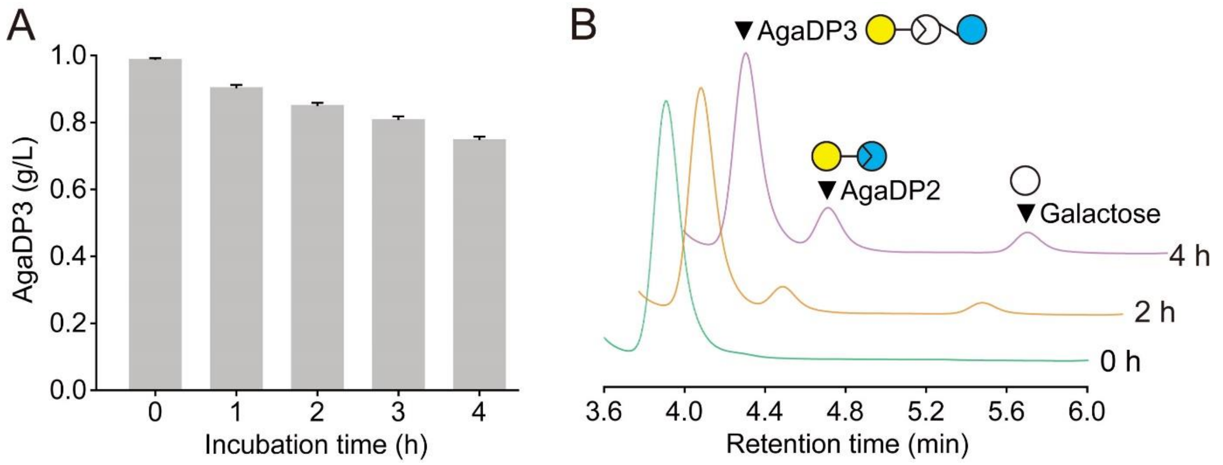

Figure S2. Stability test of AgaDP3 in the presence of simulated gastric fluid. (**A**) AgaDP3 was incubated with simulated gastric fluid comprising 0.2% (w/v) sodium chloride in 0.7% (v/v) hydrochloric acid at 37°C for 3 h. The concentration of AgaDP3 was monitored using HPLC. (**B**) Overlaid HPLC chromatograms profiling the partial degradation of AgaDP3 during incubation.

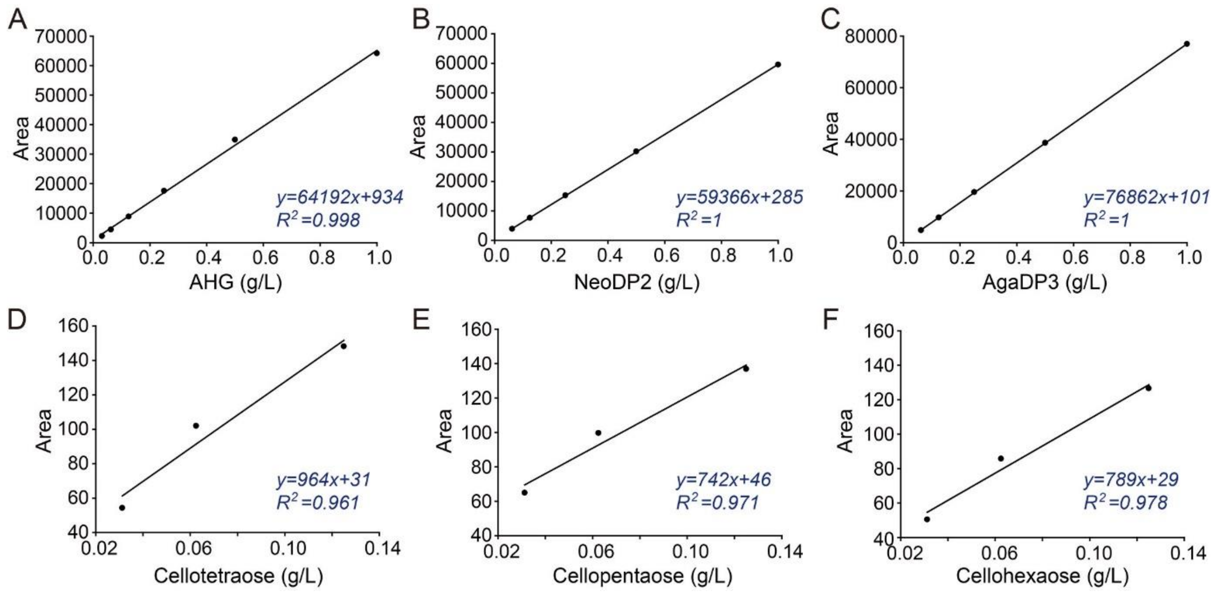

Figure S3. Calibration curves of purified agar-derived sugars produced from agarose by the enzymatic reactions of Aga16B, Aga50D, and *Sd*NABH. (**A**–**C**) Calibration curves of AHG, NeoDP2, and AgaDP3 for quantitative analyses by HPLC. (**D**–**F**) Calibration curves for cellotetraose, cellopentaose, and cellohexaose for quantitative analyses of NeoDP4, AgaDP5, and NeoDP6, respectively, by HPAEC-PAD.
